# Persistent financial hardship, 11-year weight gain, and health behaviors in the Whitehall II study

**DOI:** 10.1002/oby.20875

**Published:** 2014-08-25

**Authors:** Annalijn I Conklin, Nita G Forouhi, Eric J Brunner, Pablo Monsivais

**Affiliations:** 1 MRC Epidemiology Unit, University of Cambridge, Institute of Metabolic ScienceAddenbrooke's Hospital, Cambridge, UK; 2 UK Clinical Research Collaboration Centre for Diet and Activity Research, MRC Epidemiology Unit, University of Cambridge, Institute of Metabolic ScienceAddenbrooke's Hospital, Cambridge, UK; 3 Department of Epidemiology and Public Health, University College LondonLondon, UK; 4 Department of Public Health and Primary Care, Institute of Public Health, University of CambridgeUK

## Abstract

**Objective:**

To ascertain prospectively gender-specific associations between types and amounts of financial hardship and weight gain, and investigate potential behavioral mechanisms.

**Methods:**

Prospective study of 3701 adult British civil servants with repeated measures of difficulty paying bills or insufficient money to afford adequate for food/clothing (1985-1988; 1989-1990; 1991-1993; 1997-1999), and weight (1985-1988; 1997-1999).

**Results:**

Persistent hardships were associated with adjusted mean weight change in women over 10.9 years, but no consistent pattern was seen in men. During follow-up, 46% of women gained ≥5 kg. Women reporting persistent insufficient money for food/clothing had a significantly greater odds of gaining ≥5 kg (1.42 [1.05, 1.92]) compared to no hardship history, which remained after socioeconomic status (SES) adjustment (1.45 [1.05, 2.01]). The association between persistent difficulty paying bills and odds of excess weight gain was also significant (1.42 [1.03, 1.97]) but attenuated after considering SES (1.39 [0.98, 1.97]). Four health behaviors as single measures or change variables did not attenuate associations.

**Conclusions:**

Results suggested strategies to tackle obesity must address employed women's everyday financial troubles which may influence weight through more biological pathways than classical correlates of economic disadvantage and weight.

## Introduction

A well-developed literature has associated overweight and obesity with socioeconomic status (SES). In high-income countries, overweight and obesity tend to be more prevalent in deprived regions and among populations with lower income, educational attainment and social class 1,2. Beyond SES, more recent research has suggested that financial hardships (FH) are also strongly related to obesity, with associations that are often stronger than those reported for conventional measures of SES 3–6.

Published work on financial hardship and adiposity has been limited by a number of factors. Only two prospective studies examined the independent link between cumulative hardship and obesity in adulthood 5,7, and, with one exception 6, all relevant studies used self-reported measures 4,5,7,8. Great scope exists to further examine the impact on measured weight from different types of FH and especially to understand whether and how women and men differ in vulnerability or strength of impact. In addition, there is a need to understand the pathways by which SES, or FH, might influence adiposity. Research on potential mediators between SES and adiposity has focused on lifestyle factors 2,9,10. For example, smoking was associated with low SES, lower BMI and lower rates of weight gain 11,12. While smoking and other lifestyle factors were further patterned by gender, few investigations have explicitly conducted gender-specific mediation 2. The role of potential mediators is absent from current literature on FH and adiposity, although a range of mechanisms are likely to contribute and vary by gender 13.

In this prospective study, we examined overall associations between two types, and amounts, of FH and measured weight change over 11 years in employed middle-aged women and men in Britain, while considering conventional SES measures. As individuals can experience transitions in health behaviors associated with both cumulative hardship and excess weight gain, the study also investigated whether change in diet, physical activity, smoking, and alcohol consumption contributed to any excess weight gain.

## Methods

### Study population

This study used data from the Whitehall II study—a cohort of London-based civil servants aged 35-55 (*n* = 10,308) working in 20 departments 14. Repeated postal questionnaires provided exposure data on cumulative FH (1985-1988; 1989-1990; 1991-1993; 1997-1999). Cohort participants who responded to hardship questions once or more over the study period (*n* = 6221) showed a similar sociodemographic profile to participants responding at baseline (*n* = 6429) (Table S1). Two clinical examinations (1985-1988; 1997-1999) provided adiposity outcome data (*n* = 5704). The available sample included participants who had data on FH, covariates, and anthropometry (range: 3671-3701). All volunteers gave written informed consent and the study was approved by the University College London ethics committee.

### Measures

#### Cumulative hardship exposures

Two self-reported questions assessed FH, according to Pearlin's list of chronic strains for household economics 15. These covered *frequency of not having enough (hereafter, “insufficient”) money to afford adequate food or clothing* (five responses, “never” to “always”), and *difficulty in meeting payment of bills* (six responses, “none” to “very great”). Responses “always”, “often”, and “sometimes”, or “very great”, “great”, and “some”, were combined to construct a binary variable to indicate exposure at each time point. Dichotomized variables contributed to a 3-level dose variable of cumulative hardship, comprising a reference group (not exposed at any time point), occasional hardship (exposed at one time point) and persistent hardship (exposed at ≥2 time points).

#### Adiposity outcomes

Weight (kg) was measured using standardized protocols in clinical examinations 16. Baseline weight was subtracted from follow-up weight to calculate weight change for each participant. As weight change encompasses gain, loss, and no change, we also examined excess weight gain using WHO's threshold of ≥5 kg during adulthood for increased risk in chronic conditions 17. Hence individuals were classified as either gaining ≥5 kg, or not, over follow-up.

#### Covariates

Covariates included baseline weight, follow-up duration (years), ethnicity (binary), and, for overall associations, mid-point age (continuous), current smoker (binary) and married/cohabiting (binary). Conditioning on SES considered three conventional measures: baseline education, and midpoint employment grade and home ownership (all categorical).

#### Data analysis

Descriptive statistics summarized socio-demographic and health characteristics and adiposity outcomes across levels of cumulative hardship. A correlation coefficient matrix assessed the inter-relationship of the two FH measures. The *a priori* strategy for main analyses was to examine gender-specific associations of both types of cumulative hardship in relation to subsequent weight change, or excess weight gain, independent of SES. Thus, each cumulative hardship variable was examined separately in linear or logistic regression models using a cross-product term for gender and the exposure, with significant gender difference set at *P* < 0.05. Resulting coefficients of linear regressions were then used for post-estimation of gender-specific adjusted means and 95% confidence intervals (CI95). Analyses conditioned on baseline weight, follow-up years, ethnicity, age, current smoking, and being married (Model A). Model A additionally included all three conventional SES indicators (Model B). Results are presented as adjusted means, or odds ratios, and CI95.

In analyses of excess weight gain, we progressively adjusted for four behavioral factors that are strongly correlated with both SES and obesity. We examined factors measured at midpoint (1991-1993) and also change in each factor. Data on smoking behavior at baseline and follow-up were dichotomized to construct a change variable by defining four categories of persistent/never/initiating/stopping smokers. A continuous change variable was calculated for dietary intakes of total energy (kcal/d) and alcohol (units/week) available from midpoint and follow-up Food Frequency Questionnaires. Finally, questions at baseline and midpoint on moderate and vigorous physical activity were combined as MVPA (≥1 h/week) 18 and dichotomized to then assess change in MVPA.

Sensitivity analyses of overall independent associations between cumulative hardship and adiposity excluded baseline weight, or included additional confounders. Information on other confounders from midpoint General Health Questionnaire included: self-rated general health status (categorical), and depression and anxiety subscales (linear). Independent associations with mean weight change were examined separately for women's menopause age. Independent associations with excess weight gain were also adjusted for baseline height. Robust variance estimates were computed to test for potential clustering by civil service department, with no alteration of results. Statistical analyses were conducted using Stata 12.1.

## Results

Study follow-up averaged 10.9 years (SD 0.6), with participants averaging 44 years (SD 6) at baseline. The sample comprised nearly 30% women (*n* = 1042), 8% non-white, and 31% educated up to age 16. Lowest education level differed by gender (27% of men vs. 41% of women). The lowest occupational status comprised 17% of the sample; again, more of the women (42%) were in this group than men (6%). By midpoint, they were generally in good-to-excellent general health (90%), not depressed (87%), married/cohabiting (78%), and not current smokers (87%). Over the follow-up period, average weight change was 4.3 kg (SD 5.7) in men and 5.0 kg (SD 7.0) in women. Excess weight gain (≥5 kg) occurred in 42% of men and 46% of women. The two types of hardship were moderately related: frequency of insufficient money for food/clothing shared 31% of its variability with difficulty paying bills (*r* = 0.69).

Nearly one-fifth of respondents reported persistent insufficient money for food/clothing (16%) or persistent difficulty paying bills (18%). Table1 below showed that both types of cumulative hardship were closely related to several sociodemographic measures. Excess weight gain was more prevalent among participants reporting persistent hardships compared to those reporting no history.

**Table 1:** Sociodemographic and health characteristics of participants in the Whitehall II study across levels of cumulative financial hardship

	Mean age	Women	Non-white	Not married	Current smoker	Lowest education^a^	Lowest occupational status^b^	Non-owner^c^	Poor/ fair health^d^	Depressed^e^	Mean weight change (kg)	Gain of ≥5 kg
**History of insufficient money to afford adequate food/clothing (*n* = 3701)**												
**None *(n* = *2361)***	50 (6)	28%	5%	21%	10%	29%	11%	5%	7%	10%	4.3 (6)	41%
**Occasional *(n* = *659)***	49 (6)	27%	10%	22%	16%	34%	15%	7%	13%	14%	4.5 (6)	44%
**Persistent *(n* = *681)***	48 (6)	32%	17%	24%	18%	36%	27%	11%	16%	20%	5.2 (7)	49%
**History of difficulty paying bills (*n* = 3671)**												
**None *(n* = *2509)***	50 (6)	29%	6%	22%	11%	30%	12%	5%	7%	10%	4.3 (6)	41%
**Occasional *(n* = *586)***	49 (6)	26%	9%	21%	15%	33%	13%	6%	13%	15%	5.1 (6)	47%
**Persistent *(n* = *576)***	49 (6)	30%	14%	24%	20%	31%	25%	12%	16%	22%	4.8 (7)	48%

	Mean energy intake (kcal)	Mean change in energy (kcal)^f^	Physically active	Remained physically active^g^
**History of insufficient money to afford adequate food/clothing (*n* = 3701)**				
**None *(n* = *2361)***	2124 (601)	110 (580)	21%	11%
**Occasional *(n* = *659)***	2097 (717)	141 (664)	21%	10%
**Persistent *(n* = *681)***	2063 (673)	81 (680)	18%	8%
**History of difficulty paying bills (*n* = 3671)**				
**None *(n* = *2509)***	2105 (625)	128 (584)	21%	11%
**Occasional *(n* = *586)***	2126 (637)	60 (654)	21%	12%
**Persistent *(n* = *576)***	2119 (690)	99 (683)	20%	10%

Time points for measurement of variables: sex, education, ethnicity (1985-1988); age, marital status, smoking status, moderate and vigorous physical activity (MVPA), energy intake, self-rated general health, self-reported depression, occupational status, home ownership (1991-1993); and weight change or gain (1985-1999).

a Lowest education category, of three groups, included participants educated to age 16.

b Lowest occupation status, of three categories, was clerical/support.

c Nonowner included participants reporting they lived in accommodation rented from councils (public), privately and furnished, or privately and unfurnished.

d Self-rated general health was reported in the General Health Questionnaire (GHQ) on a 5-point scale (excellent/very good/good/fair/poor).

e Depression was assessed by the GHQ, and participants were classified as “depressed” when they scored ≥4 (score range 0-12).

f A delta variable for change in energy intake (kcal) was calculated by subtracting kcal data available at midpoint from kcal data at follow-up; kcal data was a derived variable based on Food Frequency Questionnaires administered at both time points.

g Baseline and midpoint questionnaire data on MVPA (≥1 h/week) were combined to derive a binary indicator at each time point which was then used to construct a change variable with four possible categories (persistent/never/initiating/stopping physically active).

### Cumulative hardship and mean weight change

**Figure 1:**
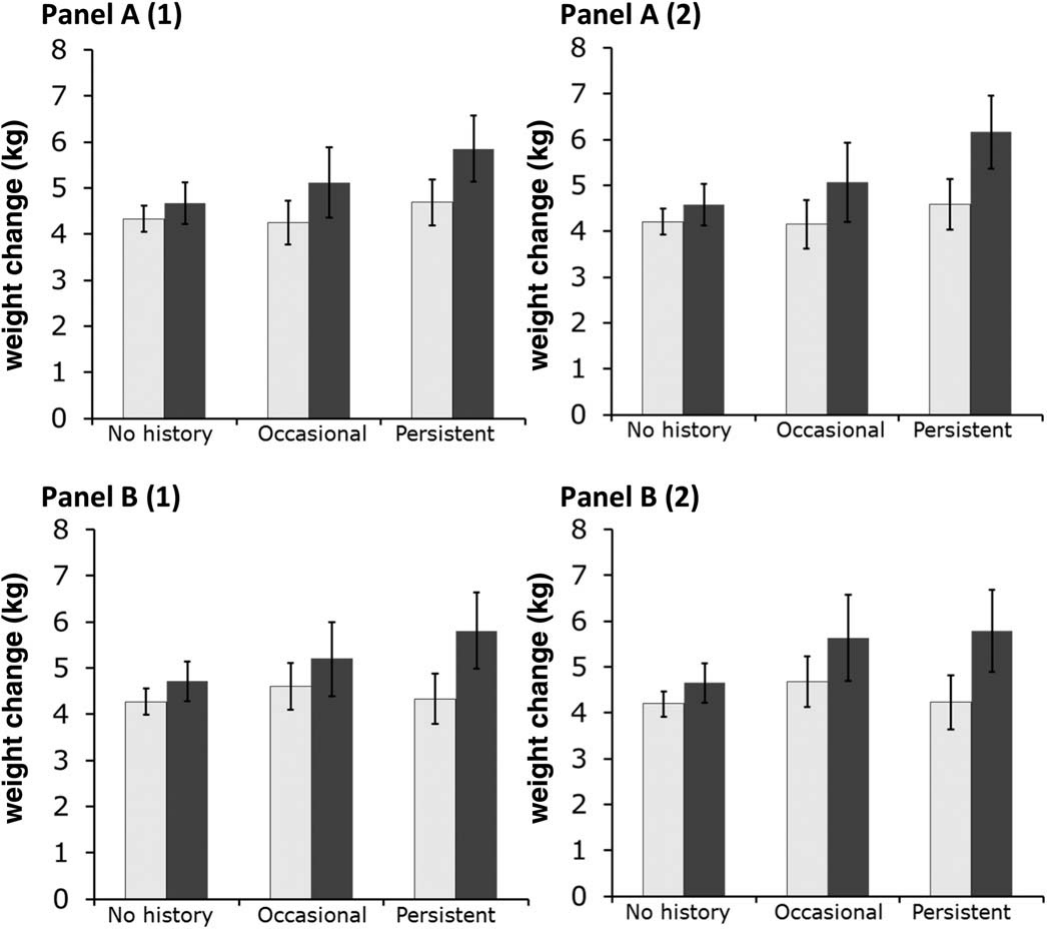
Adjusted mean 11-year weight change in women and men and cumulative financial hardship in the Whitehall II study. Men, white bars; women, black bars. (A) History of frequently insufficient money for food/clothing. (B) History of difficulty paying bills. Gender-specific results obtained by multivariable linear regression analysis adjusting for baseline weight, follow-up years, ethnicity, midpoint age, current smoker, and married (A–1, B–1), and then also for SES (A–2, B–2). Numbers were: insufficient money for food/clothing (A–1=4,025; A–2=3,701); difficulty paying bills (B–1=3,923; B–2=3,671).

Prospective analyses showed a significant association between persistent FH of both types and 11-year weight change in women only (Figure 1). Compared to women reporting no history of insufficient money for food/clothing for whom weight changed to +4.67 kg (4.22-5.12), adjusted mean weight change in women reporting persistent insufficient money for food/clothing was significantly greater (+5.85 kg [5.13, 6.57]) (Panel A-1). SES adjustment strengthened this association to +6.17 kg (5.37, 6.96) (Figure 1, Panel A-2), and revealed a significant linear trend (*P* = 0.025) and difference from men (*P* = 0.048). Adjusted mean weight also changed in women reporting persistent difficulty paying bills (+5.81 kg [4.98, 6.64]) (Panel B-1), even after SES adjustment (+5.79 kg [4.89, 6.68]) (Panel B-2).

Sensitivity analyses excluding baseline weight or including physical and mental health minimally altered the results (Table S2); significant associations also remained after computing robust variance estimates (Table S3). Women's menopause age minimally reduced mean weight change across histories of hardship (range: 0.43-0.99 kg), but increased differences between the extremes by 0.14 kg.

### Cumulative hardship and excess weight gain

Compared with no history, women reporting persistent insufficient money for food/clothing over 11 years had greater odds of gaining ≥5 kg (1.42 [1.05, 1.92]) (Table2, Model A). The statistically significant association was similar after SES adjustment (1.45 [1.05, 2.01]) (Table2, Model B). Persistent difficulty paying bills (reference: no history) also increased women's likelihood of excess weight gain by 42% (1.03, 1.97), but was attenuated after SES adjustment (1.39 [0.98, 1.97]). Results of sensitivity analyses for excess weight gain showed little change to observed associations (Table S4-S6).

**Table 2:** Odds of excess weight gain in women and men and cumulative financial hardship in the Whitehall II study

	*Model A*	*Model B: A + SES*
Women		
**History of insufficient money for food/clothing**		
**None**	1.00	1.00
**Occasional**	0.95 (0.70, 1.30)	1.01 (0.72, 1.42)
**Persistent**	1.42 (1.05, 1.92)	1.45 (1.05, 2.01)
**History of great difficulty paying bills**		
**None**	1.00	1.00
**Occasional**	1.12 (0.81, 1.54)	1.26 (0.88, 1.81)
**Persistent**	1.42 (1.03, 1.97)	1.39 (0.98, 1.97)
Men		
**History of insufficient money for food/clothing**		
**None**	1.00	1.00
**Occasional**	1.06 (0.87, 1.29)	1.03 (0.83, 1.28)
**Persistent**	1.15 (0.94, 1.41)	1.13 (0.91, 1.41)
**History of great difficulty paying bills**		
**None**	1.00	1.00
**Occasional**	1.06 (0.86, 1.30)	1.09 (0.88, 1.36)
**Persistent**	1.11 (0.90, 1.38)	1.08 (0.86, 1.36)

Gender-specific odds ratios (CI95) of gaining ≥5 kg obtained by multivariable logistic regression analysis adjusting for baseline weight, follow-up years, ethnicity, midpoint age, current smoker, and married (Model A), and then also for SES (Model B). Numbers were: insufficient money for food/clothing (Model A: 4025; Model B: 3701); difficulty paying bills (Model A: 3923; Model B: 3671).

### The role of behavioral factors

In covariate- and SES-adjusted models of excess weight gain, the progressive adjustment for single measures of dietary energy and alcohol intakes as well as MVPA did not alter significant overall associations in women (Table3). Persistent difficulty paying bills reached significance after all covariates, SES and additional behavioral factors were examined (1.43 [1.01, 2.04]) (Table3, Model 4). But since hardship accumulating over 11 years might change behavioral factors that in turn could affect excess weight gain, we also re-examined associations with progressive adjustment for change in behavioral factors (Table4). Thus, Model 1 includes all covariates and SES indicators except for current smoker which was examined as a change variable in Model 2. Progressive adjustment of change in smoking, dietary energy intake, alcohol intake and MVPA minimally amplified significant overall associations between persistent insufficient money for food/clothing and odds of excess weight gain in women.

**Table 3:** Odds of excess weight gain in women by cumulative financial hardship, with progressive adjustment for behavioral factors in the Whitehall II study

	Model 1	Model 2: 1 + dietary energy intake	Model 3: 2 + alcohol intake	Model 4: 3 + MVPA
**History of insufficient money for food/clothing**				
**None**	1.00	1.00	1.00	1.00
**Occasional**	1.01 (0.72, 1.42)	1.01 (0.72, 1.42)	1.01 (0.71, 1.43)	1.01 (0.72, 1.43)
**Persistent**	1.45 (1.05, 2.01)	1.46 (1.05, 2.02)	1.48 (1.06, 2.05)	1.49 (1.07, 2.06)
**History of difficulty paying bills**				
**None**	1.00	1.00	1.00	1.00
**Occasional**	1.26 (0.88, 1.81)	1.27 (0.88, 1.82)	1.26 (0.88, 1.81)	1.27 (0.88, 1.82)
**Persistent**	1.39 (0.98, 1.97)	1.41 (0.99, 2.00)	1.42 (1.00, 2.02)	1.43 (1.01, 2.04)

Odds ratios (CI95) of gaining ≥5 kg obtained by multivariable logistic regression analysis adjusting for baseline weight, follow-up, ethnicity, midpoint age, current smoker, married, and SES (Model 1). Numbers were: insufficient money for food/clothing (Model 1: 3701; Model 2: 3678; Model 3: 3678; Model 4: 3678); difficulty paying bills (Model 1: 3671; Model 2: 3647; Model 3: 3647; Model 4: 3125).

**Table 4:** Odds of excess weight gain in women by cumulative financial hardship, with progressive adjustment for change in behavioral factors in the Whitehall II study

	Model 1	Model 2: 1 + change in being a current smoker	Model 3: 2 + change in dietary energy intake	Model 4: 3 + change in alcohol intake	Model 5: 4 + change in MVPA (exercise)
**History of insufficient money for food/clothing**					
**None**	1.00	1.00	1.00	1.00	1.00
**Occasional**	1.02 (0.73, 1.43)	1.03 (0.74, 1.46)	0.87 (0.59, 1.27)	0.86 (0.59, 1.27)	0.86 (0.59, 1.27)
**Persistent**	1.41 (1.03, 1.94)	1.44 (1.04, 1.98)	1.47 (1.03, 2.10)	1.49 (1.04, 2.13)	1.51 (1.05, 2.17)
**History of difficulty paying bills**					
**None**	1.00	1.00	1.00	1.00	1.00
**Occasional**	1.26 (0.89, 1.79)	1.26 (0.88, 1.80)	1.27 (0.86, 1.87)	1.27 (0.85, 1.88)	1.24 (0.83, 1.84)
**Persistent**	1.36 (0.97, 1.92)	1.40 (0.99, 1.98)	1.43 (0.96, 2.11)	1.44 (0.97, 2.13)	1.46 (0.98, 2.18)

Odds ratios (CI95) of gaining ≥5 kg obtained by multivariable logistic regression analysis adjusting for baseline weight, follow-up, ethnicity, midpoint age, married, and SES (Model 1). Numbers were: insufficient money for food/clothing (Model 1: 3775; Model 2: 3732; Model 3: 3156; Model 4: 3149; Model 5: 3122); difficulty paying bills (Model 1: 3732; Model 2: 3691; Model 3: 3131; Model 4: 3125; Model 5: 3097).

## Discussion

This prospective study found women to be more vulnerable to long-term weight change, and excess weight gain, from cumulative exposure to financial hardship, independent of SES. Women reporting persistent insufficient money for food/clothing gained 1.59 kg more than women with no history of this hardship, over approximately 11 years. Similarly, women experiencing persistent difficulty paying bills gained 1.14 kg more than those reporting no such hardship. Moreover, women reporting persistent insufficient money for food/clothing had a 45% greater likelihood of excess weight gain compared to those without hardship. Men showed no differential weight change or gain in excess weight across levels of cumulative hardships. Adjustment for diet, physical activity, smoking and alcohol consumption had no impact on the results, either when these health behaviors were operationalized as a one-time measure or when they were operationalized as change variables.

### Relationship to previous work

That persistent hardship showed independent associations with adiposity is consistent with other predominantly cross-sectional studies 4–8. Independent associations between overall financial hardship and odds of gaining ≥5 kg in self-reported weight were observed in middle-aged female employees in Finland (OR range 1.50-1.70), but cumulative exposure was not measured, and living arrangement and key lifestyle factors related to adiposity not considered 5. Prolonged hardship over 1 year was examined in the Australian population, independent of income or education, and increased the risk of obesity measured a year later by 20%, more so than income 7. More broadly, cumulative financial stress had a dose-response effect on several health outcomes in Swedish women, but was less consistently related to men's outcomes 19. Similarly, more years in poverty (a ratio of income-to-theoretical needs) monotonically reduced self-rated health in US adults 20, and income-based measures of sustained hardship had a strong graded effect on depression and some other outcomes in older American women and men 21.

Some previous work suggests independent associations are stronger in men 4,5, but this study found significant associations in women for both hardship types, particularly persistent insufficient money to afford adequate food/clothing. These gender-specific findings are consistent with a wider body of evidence: difficulty paying bills was associated with obesity in female but not male youth 8; food insecurity increased the odds of 1-year weight gain for women only 22; and women report higher impact and slower adaptation to adverse life events (e.g. job loss) associated with weight 23,24. However, the pattern of gender differences in associations of financial hardship with obesity may depend on which type of hardship or anthropometric outcome is studied. In British older adults, the independent odds of general and central obesity in women, and general obesity in men, was highest for greatest difficulty paying bills; but the independent odds of central obesity in men was highest for a different type of hardship 6. Beyond the role of income-earner, women's gender means they are more likely than men to fulfill additional roles related to bearing and raising children and also giving care and support to family members such as ill parents. Thus, women and men will be dissimilarly situated in the same employment, with women potentially having higher levels of stress and/or poor sleep because of their greater number of roles and demands on their time. The context of women's lives might therefore suggest that persistent financial hardship is a chronic stressor that exhausts women's coping repertoires, resulting in role overload 25. Finally, gender roles of women may also be implicated in our specific finding that cumulative hardship related to affording adequate food or clothing remained significantly associated with adiposity after conditioning on SES. Women are often given the gendered role of the family's food and clothing provider 26; hence persistent hardship related to this domain would be disproportionately stronger for women as it threatens their ability to fulfill their role obligations.

Although mediators of the SES-obesity association were examined in some studies 9,10,27, the present study is the first to our knowledge to explore potential mechanisms of cumulative hardship associated with adiposity, focusing on four health behaviors. Accounting for all four behaviors did not produce the expected attenuation of the relationship between persistent hardship and excess weight gain. Lack of attenuation may be because of measurement error and/or limitations of the construction of change variables which likely missed several changes which an individual may have experienced over the long follow-up period. Nevertheless, our findings were similar to null results of behavioral factors mediating the association between conventional SES and weight gain in an EPIC-Norfolk study 27. A cross-sectional study of civil servants in Britain and Finland also showed negligible or small effects of behavioral factors and living arrangement in the relationship between financial hardship and physical functioning 28.

In considering other putative mediators, it may be that prolonged financial worries led to unhealthy adiposity through biological mechanisms related to stress and inadequate sleep. Both chronic stress and insufficient sleep have independent associations with obesity 6. Objective indicators of stress and sleep patterns should be accounted for in future studies so as to examine physiological mechanisms of influence between persistent hardship and long-term weight gain. Meanwhile, prevention of excess weight gain would benefit from greater attention to employed women's experiences of different types and amounts of FH, separate from their education, occupational status and wealth. Strategies might focus on helping their management of money and budgets 7, and on improving reach of existing financial assistance programs 29.

### Methodological considerations

Self-reported exposure to FH may be subject to reporting bias, and interpretation of its meaning can also vary widely across the population. Equivalent levels of financial strain can be perceived and experienced as a normative status of daily living for some groups but as deprivation for others 30. Precedent exists, however, for the measures used here as findings of independent associations are consistent with studies of other outcomes in this cohort 18,28,31. Misclassification of exposures from reporting bias would be nondifferential as it was unlikely related to measured weight and hence would have biased results towards the null. Another source of bias is nonresponse from those in lower occupational class who may be more likely to experience cumulative hardships and be overweight. Furthermore, this cohort largely comprised employed adults in the British civil service which potentially limits generalizability of findings, although similar associations were observed in a population-based UK cohort 6.

Our findings may also be subject to residual confounding from income, which was collected after our study period. However, income is inconsistently associated with weight status or change among adults 2,32. We should note that in accounting for conventional SES indicators, our models included adjustment for employment grade which represented a wide range of salary bands 14. Our observed associations may also be confounded, or mediated, by other unobserved factors including parity 33, sleep 34 and stress 35. Finally, measurement error of self-reported exposures and behaviors from large intra-individual variation or inaccurate instruments can either increase or decrease observed associations 18,36, and thus might partly explain the amplified odds of excess weight gain from including health behaviors.

Notwithstanding these limitations, this study had a number of strengths. These include: a longitudinal design with a sufficient interval to assess change, measured weight, and adjustment for multiple SES indicators and known confounders of adiposity. This work is especially novel in several ways. First, it examined separate hardship measures to provide unique information on how different types of this economic domain might be associated with adiposity 37, thus pointing to targets for intervention. As different types of hardship can arise for diverse reasons with differential impact on body weight, the study clearly contributes to the limited evidence on effects of item-specific FH 6–8. Second, it is the first prospective study of obesity and FH to employ a gender perspective which is important because women report greater exposure to, and strength of impact from, economic disadvantages 23,24. And third, it is also the first to explore potential behavioral mechanisms underlying independent gender-specific associations of cumulative hardship and weight gain.

## Conclusion

Employed British women reporting persistent insufficient money for food or clothing were more likely to gain ≥5 kg over 11 years, independent of SES. The independent association of cumulative hardship and excess weight gain was not explained by classical correlates of economic disadvantage and weight. Results suggested that public health policy and practice standards in obesity prevention or management need to consider more than SES and address in particular employed women's greater vulnerability to prolonged financial concerns. Scope exists for research to better understand behavioral and biological mechanisms that link cumulative hardship with excess weight gain.
