# Supplementary material for: Persistent financial hardship, 11-year weight gain, and health behaviors in the Whitehall II study
Source: Obesity (Silver Spring). 2014 Aug 25;22(12):2606–12. doi: 10.1002/oby.20875 (PMC4236257; doi:10.1002/oby.20875)
Supplement: Supplementary file 1 — Supplementary Information Tables [file oby0022-2606-sd1.docx]

Table S1. Baseline socio-demographic characteristics of Whitehall II cohort participants who reported presence or absence of financial hardship at baseline and over 11 years of follow-up

|  | **Cohort at baseline** | |  | **Cohort followed up** | |
| --- | --- | --- | --- | --- | --- |
|  | **Hardship^1^** | **No hardship^1^** |  | **Hardship^2^** | **No hardship^2^** |
| ***Hardship exposure – frequency of not having enough for food/clothing*** | *N=1339* | *N=5090* |  | *N=2576* | *N=3645* |
| Age, mean (SD) | 44 (6) | 45 (6) |  | 44 (6) | 45 (6) |
| Women | 32% | 30% |  | 32% | 30% |
| Not married | 26% | 24% |  | 25% | 23% |
| Lowest education (<=16) | 38% | 32% |  | 37% | 31% |
| Lowest class (clerical) | 30% | 18% |  | 30% | 16% |
| Non-owner | 15% | 9% |  | 15% | 8% |
| Long-standing illness | 35% | 30% |  | 34% | 30% |
| Depressed | 18% | 12% |  | 16% | 11% |
| Current smoker | 21% | 16% |  | 20% | 14% |
| Not physically active | 84% | 81% |  | 82% | 81% |
| Weight, mean (SD) | 74 (13) | 73 (12) |  | 73 (12) | 73 (12) |
| Total alcohol,^3^ mean (SD) | 10.4 (14) | 12 (15) |  | 10 (14) | 13 (14) |
| ***Hardship exposure – difficulty paying bills*** | *N=1346* | *N=5085* |  | *N=2231* | *N=3856* |
| Age, mean (SD) | 44 (6) | 45 (6) |  | 44 (6) | 45 (6) |
| Women | 29% | 31% |  | 31% | 31% |
| Not married | 24% | 25% |  | 24% | 24% |
| Lowest education (<=16) | 34% | 32% |  | 34% | 32% |
| Lowest class (clerical) | 25% | 19% |  | 26% | 17% |
| Non-owner | 14% | 9.5% |  | 14% | 9% |
| Long-standing illness | 35% | 30% |  | 35% | 30% |
| Depression | 18% | 12% |  | 18% | 11% |
| Current smoker | 21% | 16% |  | 21% | 14% |
| Not physically active | 82% | 82% |  | 81% | 82% |
| Weight, mean (SD) | 75 (13) | 73 (12) |  | 74 (12) | 73 (12) |
| Total alcohol,^3^ mean (SD) | 12 (15) | 12 (14) |  | 12 (15) | 12 (14) |

^1^ Responses at baseline (1985-88) to two questions on financial hardship, with hardship defined by responses ‘always’, ‘often’, and ‘sometimes, or ‘very great’, ‘great’, and ‘some’.

^2^ Respondents at follow-up who provided data to construct a 3-level dose variable for cumulative financial hardship which comprised a reference group (no hardship at all 4 time-points), occasional hardship (hardship at any time-point) and persistent hardship (hardship at ≥2 time-points).

^3^ Total alcohol intake (units/week) from FFQ was first available at mid-point.

Table S2. Sensitivity analysis of excluding baseline weight or including additional confounders in the independent associations of cumulative financial hardship with adjusted mean weight change in middle-aged adults in the Whitehall II study

|  | **Women** | |
| --- | --- | --- |
|  | ***Model 1: Excluding baseline weight*** | ***Model 2: Including additional confounders*** |
| **History of insufficient money for food/clothing** | | |
| None | 4.57 (4.12, 5.02) | 4.60 (4.15, 5.05) |
| Occasional | 5.05 (4.18, 5.91) | 5.02 (4.16, 5.88) |
| Persistent | 6.22 (5.42, 7.01) | 6.15 (5.35, 6.95) |
| **History of great difficulty paying bills** | | |
| None | 4.65 (4.22, 5.08) | 4.67 (4.24, 5.10) |
| Occasional | 5.62 (4.69, 6.56) | 5.57 (4.64, 6.51) |
| Persistent | 5.82 (4.92, 6.71) | 5.77 (4.87, 6.67) |
|  | **Men** | |
| **History of insufficient money for food/clothing** | | |
| None | 4.20 (3.92, 4.49) | 4.22 (3.94, 4.51) |
| Occasional | 4.15 (3.62, 4.69) | 4.12 (3.59, 4.65) |
| Persistent | 4.62 (4.07, 5.17) | 4.54 (3.98, 5.10) |
| **History of great difficulty paying bills** | | |
| None | 4.18 (3.91, 4.46) | 4.20 (3.92, 4.48) |
| Occasional | 4.70 (4.14, 5.26) | 4.66 (4.11, 5.22) |
| Persistent | 4.25 (3.66, 4.84) | 4.17 (3.58, 4.77) |
| Gender-specific mean (CI95) weight change (kg) obtained by multivariable linear regression analysis adjusting for follow-up years, ethnicity, and midpoint age, current smoker and married, but not for baseline weight (Model 1), or adjusting for all covariates, SES and also for midpoint self-rated general health and depression, and anxiety subscales (Model 2). Numbers were: insufficient money for food/clothing (Model 1: 3,701; Model 2: 3,697); difficulty paying bills (Model 1: 3,671; Model 2: 3,667). | | |

Table S3. Robust variance estimates for the independent associations of cumulative financial hardship with adjusted mean weight change in middle-aged adults in the Whitehall II study

|  | **Women** | |
| --- | --- | --- |
|  | ***Model A*** | ***Model B: + SES*** |
| **History of insufficient money for food/clothing** | | |
| None | 4.67 (4.15, 5.19) | 4.58 (4.06, 5.10) |
| Occasional | 5.12 (4.30, 5.94) | 5.07 (4.18, 5.97) |
| Persistent | 5.85 (4.99, 6.72) | 6.17 (5.19, 7.14) |
| **History of great difficulty paying bills** | | |
| None | 4.71 (4.23, 5.19) | 4.65 (4.17, 5.14) |
| Occasional | 5.20 (4.26, 6.13) | 5.64 (4.49, 6.78) |
| Persistent | 5.81 (4.83, 6.79) | 5.79 (4.71, 6.86) |
|  | **Men** | |
| **History of insufficient money for food/clothing** | | |
| None | 4.33 (4.07, 4.60) | 4.21 (3.95, 4.47) |
| Occasional | 4.25 (3.82, 4.68) | 4.15 (3.67, 4.63) |
| Persistent | 4.69 (4.17, 5.21) | 4.59 (4.02, 5.15) |
| **History of great difficulty paying bills** | | |
| None | 4.27 (4.02, 4.52) | 4.20 (3.94, 4.45) |
| Occasional | 4.60 (4.12, 5.08) | 4.68 (4.14, 5.21) |
| Persistent | 4.33 (3.78, 4.89) | 4.23 (3.65, 4.81) |
| Robust variance estimates for gender-specific mean (CI95) weight change (Kg) obtained by STATA command vce(robust) in multivariable linear regression analysis adjusting for follow-up years, ethnicity, and midpoint age, current smoker and married (Model A), and additionally for SES (Model B). Numbers were: insufficient money for food/clothing (Model A: 4,025; Model B: 3,701); difficulty paying bills (Model A: 3,923; Model B: 3,671). | | |

Table S4. Sensitivity analysis of excluding baseline weight or including additional confounders in the independent associations of cumulative financial hardship with odds of excess weight gain in middle-aged adults in the Whitehall II study

|  | **Women** | |
| --- | --- | --- |
|  | ***Model 1: Excluding baseline weight*** | ***Model 2: Including additional confounders*** |
| **History of insufficient money to afford adequate food/clothing** | | |
| None | 1.00 | 1.00 |
| Occasional | 1.01 (0.72, 1.43) | 0.97 (0.69, 1.37) |
| Persistent | 1.52 (1.10, 2.10) | 1.41 (1.02, 1.97) |
| **History of difficulty paying bills** | | |
| None | 1.00 | 1.00 |
| Occasional | 1.28 (0.89, 1.83) | 1.21 (0.84, 1.74) |
| Persistent | 1.45 (1.02, 2.05) | 1.34 (0.94, 1.91) |
|  | **Men** | |
| **History of insufficient money to afford adequate food/clothing** | | |
| None | 1.00 | 1.00 |
| Occasional | 1.06 (0.85, 1.31) | 1.01 (0.81, 1.25) |
| Persistent | 1.18 (0.94, 1.47) | 1.09 (0.87, 1.37) |
| **History of difficulty paying bills** | | |
| None | 1.00 | 1.00 |
| Occasional | 1.13 (0.91, 1.41) | 1.08 (0.86, 1.34) |
| Persistent | 1.12 (0.89, 1.41) | 1.04 (0.83, 1.32) |
| Gender-specific odds ratios (CI95) of gaining ≥5 Kg obtained by multivariable linear regression analysis adjusting for follow-up years, ethnicity, and midpoint age, current smoker and married, but not for baseline weight (Model 1), or adjusting for all covariates, SES and also for midpoint self-rated general health and depression and anxiety subscales (Model 2). Numbers analysed were: insufficient money (Model 1: 3701; Model 2: 3,697); difficulty paying bills (Model 1: 3,671; Model 2: 3,667). | | |

Table S5. Sensitivity analysis of including baseline height in associations between cumulative financial hardship and odds of excess weight gain in middle-aged adults in the Whitehall II study

|  | **Women** | |
| --- | --- | --- |
|  | ***Model A*** | ***Model B: A + SES*** |
| **History of insufficient money to afford adequate food/clothing** | | |
| None | 1.00 | 1.00 |
| Occasional | 0.95 (0.69, 1.30) | 1.01 (0.72, 1.42) |
| Persistent | 1.41 (1.04, 1.90) | 1.44 (1.04, 1.99) |
| **History of difficulty paying bills** | | |
| None | 1.00 | 1.00 |
| Occasional | 1.11 (0.80, 1.52) | 1.25 (0.87, 1.80) |
| Persistent | 1.40 (1.01, 1.95) | 1.37 (0.96, 1.94) |
|  | **Men** | |
| **History of insufficient money to afford adequate food/clothing** | | |
| None | 1.00 | 1.00 |
| Occasional | 1.05 (0.87, 1.28) | 1.03 (0.83, 1.28) |
| Persistent | 1.14 (0.93, 1.40) | 1.13 (0.90, 1.41) |
| **History of difficulty paying bills** | | |
| None | 1.00 | 1.00 |
| Occasional | 1.05 (0.86, 1.29) | 1.09 (0.87, 1.36) |
| Persistent | 1.11 (0.89, 1.37) | 1.08 (0.86, 1.36) |
| Gender-specific odds ratios (CI95) of gaining ≥5kg obtained by multivariable logistic regression analysis adjusting for baseline height and weight, follow-up years, ethnicity, and mid-point age, current smoker, and married (Model A), and additionally for SES (education, occupational status, home-ownership) (Model B). Numbers analysed were: insufficient money (Model A: 4,024; Model B: 3,700); difficulty paying bills (Model A: 3,922; Model B: 3,670). | | |

Table S6. Robust variance estimates for the independent associations of cumulative financial hardship with odds of odds of excess weight gain in middle-aged adults in the Whitehall II study

|  | **Women** | |
| --- | --- | --- |
|  | ***Model A*** | ***Model B: A + SES*** |
| **History of insufficient money for food/clothing** | | |
| None | 1.00 | 1.00 |
| Occasional | 0.95 (0.69, 1.31) | 1.01 (0.72, 1.42) |
| Persistent | 1.42 (1.05, 1.94) | 1.45 (1.04, 2.02) |
| **History of great difficulty paying bills** | | |
| None | 1.00 | 1.00 |
| Occasional | 1.12 (0.80, 1.56) | 1.26 (0.87, 1.84) |
| Persistent | 1.42 (1.02, 1.98) | 1.39 (0.98, 1.97) |
|  | **Men** | |
| **History of insufficient money for food/clothing** | | |
| None | 1.00 | 1.00 |
| Occasional | 1.06 (0.87, 1.29) | 1.03 (0.83, 1.28) |
| Persistent | 1.15 (0.94, 1.41) | 1.13 (0.91, 1.41) |
| **History of great difficulty paying bills** | | |
| None | 1.00 | 1.00 |
| Occasional | 1.06 (0.87, 1.30) | 1.09 (0.88, 1.36) |
| Persistent | 1.11 (0.90, 1.38) | 1.08 (0.86, 1.36) |
| Robust variance estimates for gender-specific odds ratios (CI95) of gaining ≥5 Kg obtained by STATA command vce(robust) in multivariable logistic regression analysis adjusting for baseline weight, follow-up years, ethnicity, and mid-point age, current smoker and married (Model A), and additionally for SES (Model B). Numbers were: insufficient money for food/clothing (Model A: 4,025; Model B: 3,701); difficulty paying bills (Model A: 3,923; Model B: 3,671). | | |
